# The Development of a Wearable Biofeedback System to Elicit Temporal Gait Asymmetry using Rhythmic Auditory Stimulation and an Assessment of Immediate Effects

**DOI:** 10.3390/s24020400

**Published:** 2024-01-09

**Authors:** Aliaa Gouda, Jan Andrysek

**Affiliations:** 1 Institute of Biomedical Engineering, University of Toronto, Toronto, ON M5S 3G9, Canada; aliaa.gouda@mail.utoronto.ca; 2 Bloorview Research Institute, Holland Bloorview Kids Rehabilitation Hospital, Toronto, ON M4G 1R8, Canada

**Keywords:** gait training, temporal gait asymmetry, rhythmic auditory stimulation, biofeedback, wearable systems

## Abstract

Temporal gait asymmetry (TGA) is commonly observed in individuals facing mobility challenges. Rhythmic auditory stimulation (RAS) can improve temporal gait parameters by promoting synchronization with external cues. While biofeedback for gait training, providing real-time feedback based on specific gait parameters measured, has been proven to successfully elicit changes in gait patterns, RAS-based biofeedback as a treatment for TGA has not been explored. In this study, a wearable RAS-based biofeedback gait training system was developed to measure temporal gait symmetry in real time and deliver RAS accordingly. Three different RAS-based biofeedback strategies were compared: open- and closed-loop RAS at constant and variable target levels. The main objective was to assess the ability of the system to induce TGA with able-bodied (AB) participants and evaluate and compare each strategy. With all three strategies, temporal symmetry was significantly altered compared to the baseline, with the closed-loop strategy yielding the most significant changes when comparing at different target levels. Speed and cadence remained largely unchanged during RAS-based biofeedback gait training. Setting the metronome to a target beyond the intended target may potentially bring the individual closer to their symmetry target. These findings hold promise for developing personalized and effective gait training interventions to address TGA in patient populations with mobility limitations using RAS.

## 1. Introduction

For individuals with lower-limb mobility challenges, gait training typically encompasses high repetitions of specific walking and balance exercises to develop or restore a healthy gait pattern. Temporal gait asymmetry (TGA), defined as the uneven timing between the left and right step times during walking, is one of the most commonly occurring gait deficits in many populations with lower-limb mobility impairments (i.e., individuals with lower-limb amputation, Parkinson’s disease, strokes, cerebral palsy, etc.) [1,2,3,4,5]. Improving TGA is critical for achieving efficient mobility and preventing fatigue, as well as mitigating the risk of long-term joint pain and degeneration [2,6,7,8].

Since gait is largely a rhythmic movement, aligning one’s gait to an external rhythm has been found to improve several gait parameters, including speed, cadence, and temporal symmetry, as the tempo of the beat can be modulated or adjusted accordingly [9]. To improve TGA, previous studies have investigated the use of rhythmic stimulation, which involves the use of external cues, such as auditory or vibrotactile stimuli, to provide a pulsed rhythmic pattern (e.g., metronome) that encourages synchronization with the individual’s gait [9,10,11]. The timing of the stimuli can be controlled to affect and improve the temporal gait parameters (i.e., symmetry and cadence) [9,11]. For example, Roerdink et al. found that RAS-induced treadmill walking for individuals after a stroke was effective in improving temporal gait asymmetry compared at different walking speeds [10]. Furthermore, Crosby et al. first investigated inducing TGA using an ankle weight with neurotypical adults, followed by RAS, which was found to have improved TGA [9]. The subsequent study involved investigating the relation between improvements in TGA and the level of rhythmic ability of individuals following a stroke using RAS [12]. It was found that both groups (strong/weak rhythmic abilities) were able to improve TGA using RAS.

Overall, studies that have successfully elicited changes to TGA with rhythmic stimulation most commonly used constant open-loop rhythmic stimulation, where the metronome is unchanged and unaffected by the performance of the user. With such methods, the target symmetry is always set to perfect symmetry, rather than an incremental approach based on how well the individual is able to achieve temporal symmetry. However, research has indicated that optimal rehabilitation programs for improving walking abilities involve repetitive and extensive practice that is continually incremented in difficulty based on the tolerance and progress of the patient [13]. Hence, closed-loop systems that provide progressive gait training, such as an RAS-based biofeedback (BFB) system, may be beneficial in this case. Particularly, given that RAS enables control over cadence through the generation of a metronome synchronized to a specific rhythmic pattern, the integration of RAS within a BFB system holds promise for mitigating the issues of reduced speed and cadence that are associated with other BFB methods [14,15]. Additionally, by providing external cues, rhythmic stimulation allows for more automatic and subconscious control of gait [11]. This can also enhance the coupling between the sensory feedback and the motor output, which can facilitate the functioning of the central pattern generator, a neural network in the spinal cord responsible for generating rhythmic movement patterns, such as gait [16]. Encompassing such feedback into wearable systems that can be used for extensive periods of training (e.g., in the community) can lead to more effective gait training and relearning [17,18,19].

To our knowledge, closed-loop rhythmic stimulation has not been investigated as a means of treating TGA and as part of a wearable BFB system. Therefore, addressing the aforementioned gaps, the main objective of this study was to develop and evaluate a custom wearable BFB system based on inertial signals retrieved from the lower legs to provide a rhythmic auditory stimulation (RAS) targeting the stance–time symmetry ratio. This study focused on assessing three different feedback strategies, open- and closed-loop methods, using both constant and incrementally changing RAS. Furthermore, this study aimed to first test and evaluate the proposed RAS-based BFB system and strategies with able-bodied (AB) participants. This understanding is important for identifying any distinctions among strategies before evaluating such changes in individuals with mobility impairments (e.g., lower-limb prosthesis users). Since AB individuals typically exhibit temporal symmetry, this study aimed to induce TGA. The specific objectives of this study were (1) to develop a system to induce temporal gait asymmetry using RAS with the aforementioned strategies, (2) to evaluate the performance of the system and strategies in altering TGA, cadence, and walking speed, and (3) to evaluate aspects of usability.

## 2. Materials and Methods

### 2.1. Instrumentation

Participants were equipped with two systems: (1) the developed biofeedback system providing rhythmic auditory stimulation and (2) a wearable motion capture system to collect secondary gait parameters, including spatial and kinematic gait parameters.

**Figure 1:**
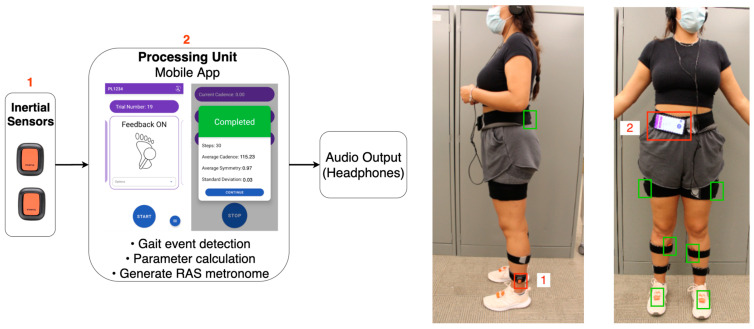
(**Left**) Flow diagram illustrating the developed wearable BFB system. (**Right**) A participant equipped with (red boxes) the developed BFB and (green boxes) wearable motion capture systems.

The developed wearable biofeedback system comprised two triaxial inertial sensors (DOT v2, Movella North America Inc., Henderson, NV, USA) connected to an Android phone via Bluetooth and a pair of headphones providing the RAS. The two Xsens DOT inertial sensors were placed on the lower legs, right above the ankle (1 on each side), as indicated in Figure 1. A custom Android mobile application was developed using Xsens DOT’s SDK and Android Studio (Google LLC, Mountain View, CA, USA). The mobile application received the streamed angular velocity and quaternion angle signals (sampled at the maximum rate of 60 Hz) from the inertial sensors, which were used for detecting key gait events, such as heel-strike (HS) and toe-off (TO), through a previously validated real-time gait event detection algorithm as presented in [20]. The application then calculated the gait parameters (e.g., stance–time symmetry ratio, cadence, and step count) and then provided the corresponding auditory metronome beat to the user, as per Figure 1. To measure TGA, a stance–time symmetry ratio (STSR) was used. The stance–time ratio was calculated as the timing difference between the TO and HS of both sides (right and left), as per Equation (1). The STSR was then calculated as the ratio of the right to left STSR, as per (2). The STSR included double-limb support time.

(1) $$ST={TO}_{i}-{HS}_{i},$$

(2) $$STSR=\frac{{ST}_{right}}{{ST}_{left}},$$

where ST represents stance time and STSR represents the stance–time symmetry ratio.

The motion capture system used was the Xsens MVN Awinda (Movella North America Inc., Henderson, NV, USA). Seven triaxial inertial sensors (±2000 deg/s, ±160 m/s^2^, ±1.9 Gauss), with a sampling frequency set to 100 Hz, were attached to the pelvis (1), upper legs (2), lower legs (2), and feet (2). Signals from the seven sensors were streamed wirelessly to a recording station, which was then connected to a laptop to save the data.

### 2.2. RAS-Based BFB Strategies

Three distinct feedback strategies using RAS were explored, as per Table 1. During constant open-loop (COL) trials, the target STSR remained constant throughout the duration of the trial. Target levels of 1.0, 0.9, 0.8, and 0.7 were all separate trials. For variable open-loop (VOL) trials, the target STSR continuously decreased by 0.3–0.4% every full gait cycle. For variable closed-loop (VCL) trials, the target STSR decreased by 3–4% only when the participant’s real-time STSR was within a range (target STSR ± error) for 80% of the time. The permissible error value used to determine the range was selected based on the participants’ baseline STSR standard deviation. The increments for VOL and VCL were selected based on pilot testing conducted to select an appropriately subtle increment (one that would not interfere with the participant’s gait unsafely).

For all three strategies, the metronome was based on a given stance–time symmetry (either pre-set or real-time) and a cadence value, as described below. To ensure that participants were able to comfortably match the cues, a cadence 5% lower than the baseline (details below) was applied during RAS. Since the protocol was designed to be tested with able-bodied individuals that had typical TGA (i.e., baseline STSR = 1.0 ± 0.05 [21,22]), the starting STSR for all three strategies was set to 1.0, and any incremental changes pushed the STSR away from symmetry (in this study, the STSR decreased to induce gait asymmetry toward the right side). By modulating AB gait, a deeper insight into the perceived level of changes induced by RAS can be obtained. While specific neuromusculoskeletal differences or biomechanical limitations may emerge based on the patient population to which these strategies are applied, the perception threshold remains transferable across populations [23].

### 2.3. Meteronome Generator

**Figure 2:**
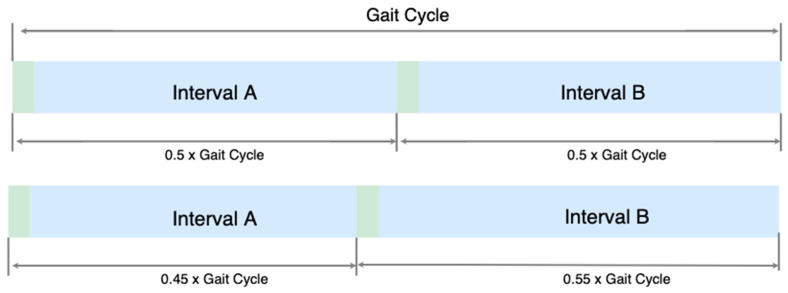
Example of the different metronomes generated based on different STSRs: (**top**) STSR = 1 and (**bottom**) STSR = 0.82. Green regions represent the duration of the beat sound (100 ms) and blue regions represent the duration with no beat sound (varying time (ms)).

A metronome generator was developed and integrated into the mobile application. The metronome’s gait cycle duration was based on the participants’ cadence calculated during the baseline trials (discussed in the experimental protocol section below). The intervals (the time between the audio beat) for each step were based on the gait cycle duration and the target STSR. Figure 2 illustrates an example of the difference between a metronome of STSR = 1 and 0.82. For VOL and VCL strategies where the target STSR was continuously updated during a trial, the metronome’s intervals would update at the end of a gait cycle.

### 2.4. Workload Assessment

To quantify the subjective outcomes of the RAS-based BFB and compare strategies, the NASA-TLX was used to assess the workload. Responses to the questionnaire were captured and processed using an HTML-based NASA TLX questionnaire developed by Keith Vertanen (NASA-TLX in HTML and JavaScript, Version). This calculates the individual ratings and weights for each of the six subscales (mental demand, physical demand, temporal demand, frustration, effort, and performance) to obtain an overall weighted mean individual score for each strategy (COL, VOL, VCL), following the procedures described by Hart et al. [24]. Scores in the ranges of 0–9, 10–29, 30–49, 50–79, or 80–100 indicate a low, medium, somewhat high, high, or very high workload, respectively [24].

### 2.5. Participants

Ten able-bodied participants (seven females and three males; 25.3 ± 8.8 years; height 170.5.9 ± 7.91 cm; weight 66.7 ± 11.7 kg) were recruited to participate in this study. Understanding the response of different types of RAS strategies in able-bodied persons is important to confirm that gait changes can be elicited in individuals with typical gait and identify any differences between strategies prior to assessing such changes with individuals with mobility impairments (i.e., lower-limb prosthesis users) [23]. A sample size of n = 10 was selected based on previously reported studies investigating wearable systems using RAS-based methods for gait training [25]. Participants were included if they were fourteen years or older and community ambulators able to walk on level ground without ambulatory aids. All participants had no previously known neurological disorders. Recruitment was facilitated through Holland Bloorview Kids Rehabilitation Hospital using posted recruitment bulletins. The study was approved (REB-0448, approved on 18 December 2021) by the Research Ethics Board at Holland Bloorview Kids Rehabilitation Hospital, Canada. Informed consent from each participant was obtained before conducting the study.

### 2.6. Experimental Protocol

**Figure 3:**
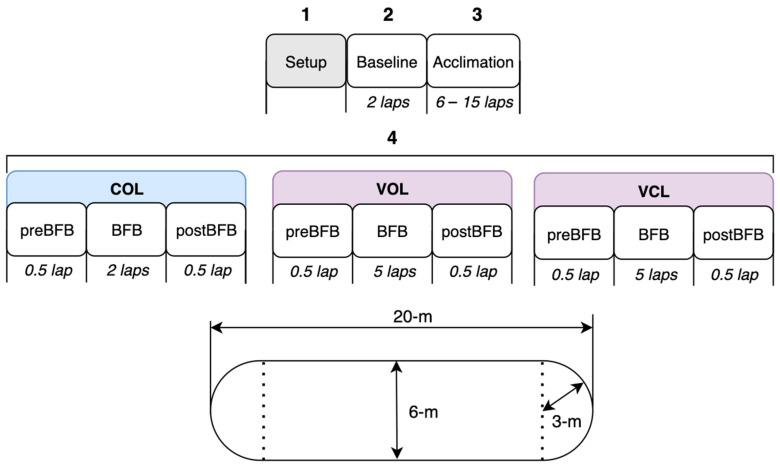
(**Top**) Outline of the sequence of tasks/trials for the sessions. (**Bottom**) Illustration of a single lap.

The data collection session involved 4 main stages: system setup, baseline, acclimation period, and gait training period, as outlined in Figure 3. (1) For the setup, participants were instrumented with the BFB and Xsens systems (Figure 1). The wearable motion capture system was calibrated using the N-pose method, where the participant remained in a neutral pose for 5 s and then walked in a straight line back and forth [26]. For the developed BFB system, the inertial sensors were strapped to the participants and connected to the mobile phone via Bluetooth. A mobile phone was also attached to the participants. Hence, only for monitoring purposes for the research coordinator, the mobile application was connected to a local server using a Python script to stream and display all the gait calculations in real time on a laptop. (2) For the baseline, participants walked 2 laps (details below), where the stance–time symmetry ratio (mean ± standard deviation), cadence, and step count were measured. (3) During the acclimation period, participants were coached about how to interpret the rhythmic stimulation; participants were instructed to associate their HS timings with the beat heard. Depending on the self-reported level of comfort, participants completed between 6 to 15 laps to become acclimated to the RAS. (4) Gait training using each strategy was conducted. The order of strategies was randomized for each participant. For COL, 4 different target levels were used in a randomized order. Hence, every 3 laps, the BFB was reset to the new target level (participants restarted the trial at each new target level). For VOL and VCL, since the target automatically changed, all the trials were continuous over 6 laps. A lap was a 20 m pass with a 3 m radius turn, as illustrated in Figure 3. The study was conducted in the gymnasium at Holland Bloorview Kids Rehabilitation Hospital.

Between each strategy, a 5 min break was provided to the participants, in which they were asked to complete the NASA Task Load Index (NASA-TLX) questionnaire relating to the strategy that they just completed. Additionally, to minimize the carry-over effect from the previous trial, participants were asked to walk for 1–2 min without listening to any metronomes.

### 2.7. Post Processing

The data from the mobile application were saved and extracted to .csv format, whereas the data from the Xsens MVN Awinda were processed and extracted using MVN Analyze software to .xml format. The main gait parameter extracted from this system was walking speed.

**Figure 4:**
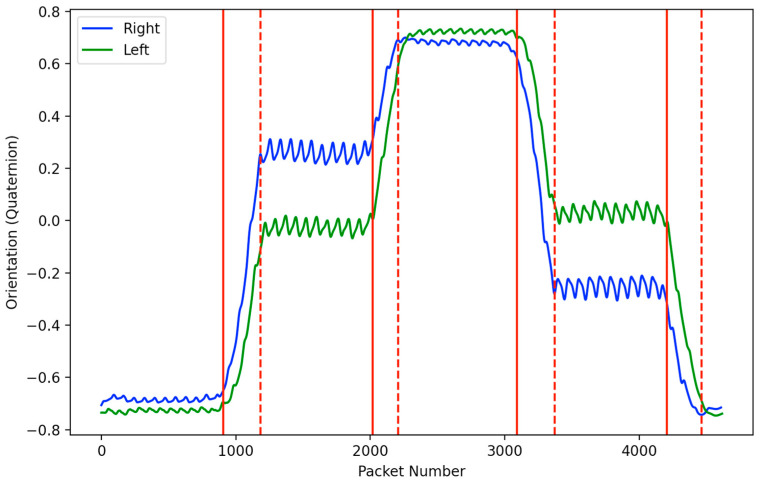
Right (blue) and left (green) orientation signals from the lower leg inertial sensors. Start (solid red) and end (dashed red) of the turn detected.

Since the trials were conducted as a sequence of continuous laps, the turning steps during each trial were excluded from the analysis using a turn detection algorithm. To identify all the strides during the turning periods, a simple heuristics algorithm was developed. The algorithm was based on the Xsens DOT sensors’ 3D orientation (quaternion angles), which is computed using their propriety Xsens sensor fusion algorithms [27]. A sliding window of 25 samples was used to identify and save all minimum and maximum values. If the difference between 2 consecutive min/max values was greater than a set threshold of 0.2, the turning state was set to 1; otherwise, it was set to 0. This was implemented for both right and left signals. Turn starts were then identified as the point when the turning state switches from 0 to 1 and vice versa for turn ends. Figure 4 illustrates the turns detected as per the aforementioned algorithm.

### 2.8. Data Analysis

Data collected from the developed mobile application included the STSR and cadence values for each gait cycle, while speed data were obtained using Xsens. To evaluate the extent of an individual’s STSR change in comparison to their baseline and target level, a percent of STSR change was calculated for target levels less than 1, following (3).

(3) $$Percent\;of\;STSR\;Change=\frac{{STSR}_{actual}-{STSR}_{baseline}}{{STSR}_{target}-{STSR}_{baseline}}\times 100\%$$

To conduct the analysis at different target levels, the data were organized into clusters based on the nearest tenth. For instance, a target STSR of 0.93 was grouped within the 0.9 cluster. To assess the normality of the distribution of data, a Shapiro–Wilk test was conducted (*p* < 0.05), indicating that the STSR data were non-parametric. Hence, a non-parametric two-tailed Wilcoxon signed-rank test was used for STSR data analysis. Speed and cadence data followed a normal distribution. Therefore, a two-tailed independent *t*-test was employed to assess significant differences. Python and the SciPy library [28] were used for statistical analysis.

Data analysis adhered to the following structure:

1. Performance of each strategy: STSR values during BFB were compared to the baseline to determine whether RAS induced any changes in the STSR for each strategy. Additionally, within each strategy, STSR values at each target level were compared to identify the most significant changes. Cadence and speed at each target level were also compared to the baseline (target STSR = 1) to detect any significant alterations;
2. Comparison of strategies: Significant differences between each strategy were analyzed at each target level. Furthermore, workload was evaluated by comparing the NASA-TLX scores for significant differences between the strategies.

## 3. Results

### 3.1. Performance of Each Strategy

**Figure 5:**
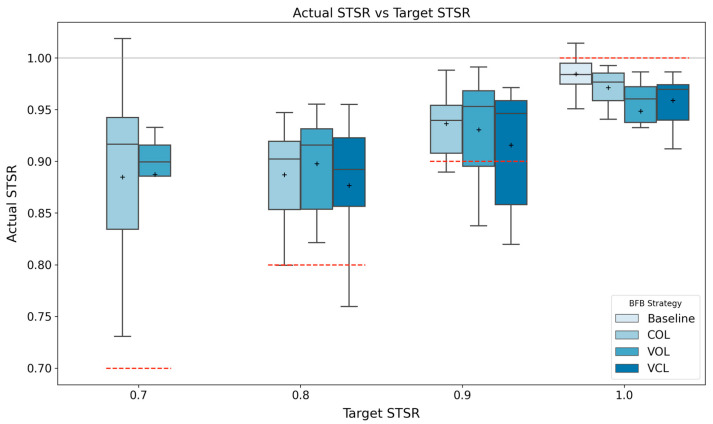
Box plot figures for each strategy at each target STSR level (dashed red) across all participants. The horizontal solid grey line indicates a perfect symmetry ratio. Black plus (+) represents mean values.

The average STSR for all the conditions is presented in Figure 5 and Table 2. For all the strategies, the mean STSRs at target levels of 0.7, 0.8, and 0.9 were significantly different compared to the baseline. The percent change values are presented in Table 3, showing how far the participants’ STSR was relative to the target and baseline values, as a percentage.

The pairwise comparison between each target level for each strategy indicated significant differences, except for between (1.0, 0.9) for VOL and VCL, (0.9, 0.8) for VOL and VCL, (0.9, 0.7) for COL, and (0.8, 0.7) for COL and VOL, as shown in Table 4. However, when observing the changes using the percent of STSR change values, no significant differences were found between each target, as per Table 4.

During all three strategies, cadence and speed did not significantly change throughout the trials, as indicated by the results in Table 5. Since the cadence was set 5% lower than each participant’s baseline, a pairwise comparison was conducted relative to the cadence and speed for COL at target level 1.

### 3.2. Comparison of Strategies

Comparing COL, VOL, and VCL to each other using the STSR and percent of STSR change values, there was no statistically significant difference between each strategy at each target level, except for VCL relative to COL and VOL at target level 0.8, as per Table 6. Additionally, to measure the response delay between the HS and audio occurrences for each side, the time error of their respective timestamps was calculated, as per Table 7. Significant differences were found between the right and left sides for COL and VOL (i.e., the left side lagged significantly more than the right side).

The results of the NASA-TLX questionnaire indicated that the overall workloads for COL, VOL, and VCL were 44.58 ± 16.22, 45.56 ± 16.26, and 49.06 ± 14.31, respectively, as outlined in Table 8. Based on the pairwise comparison (independent *t*-test) results, all three strategies exhibited similar workload demand levels. Based on the NASA-TLX score scale, the mean results are categorized as somewhat high.

## 4. Discussion

This study proposed a novel wearable RAS-based biofeedback method to elicit changes in temporal gait symmetry while also controlling for cadence and indirectly walking speed. Building on previous work, two RAS-based feedback strategies (VOL, VCL) were proposed and compared to a previously validated one (COL) [9,12,29]. Although RAS has been previously studied for eliciting gait parameter changes, to the best of the authors’ knowledge, this was the first study that applied and evaluated RAS for biofeedback gait training in a closed-loop manner.

Using all three strategies (COL, VOL, VCL), temporal gait symmetry significantly changed as the target moved further away from baseline symmetry, as illustrated in Figure 5. TGA increased as the target asymmetry moved further from the baseline symmetry, with average STSR changes ranging from 0.04 to 0.10 based on the different strategies and STSR target levels, as outlined in Table 3. Previous studies have investigated inducing TGA in neurotypical/healthy individuals using ankle weights and then eliciting symmetry using RAS, which resulted in average symmetry changes ranging between 0.09 and 0.13 [11,23]. On the other hand, Crosby et al. [12] reported averaged temporal symmetry improvements of 8% using RAS with individuals following a stroke. Similarly, Hyun et al. [29] investigated the use of RAS with individuals post-stroke and evaluated five different conditions (i.e., various strategies of aligning the metronome with gait at different walking speeds). This study reported temporal symmetry improvements ranging between 0.06 and 0.12, depending on the strategy used. Hence, the results from this study are comparable to previous studies inducing TGA. Moreover, while there were significant changes compared to the baseline, the percent of STSR change indicated that the RAS strategies did not elicit 100% change (i.e., matching the target symmetry exactly). This has previously been reported with studies using RAS to induce TGA changes, where participants did not achieve perfect temporal symmetry (i.e., STSR = 1.0) using a symmetrical metronome (i.e., target STSR = 1.0) [9,11,12].

Interestingly, when comparing the STSR data at different target levels for each strategy, the results in Table 4 indicate that there were significant differences between each target level, except for the (0.7, 0.8) comparison. This indicates that there may be a saturation level at which the RAS-based strategies no longer perturbed the participants’ TGA. Although we were not able to evaluate if the same observations are present for VCL (0.8, 0.7) since these levels were not reached, the changes in target levels were similar to COL and VOL at the other target levels (0.8, 0.9). On the other hand, comparing the percent of STSR change for target levels 0.8 and 0.9, no significant differences were found for COL and VCL strategies, as per Table 4. However, comparing to target level 0.7, significant differences were found for both COL and VOL strategies. This implies that by setting a higher asymmetry (i.e., lower target level), RAS can still elicit changes with a similar offset (as per the percent of STSR change), as observed comparing target levels 0.8 and 0.9. However, at extreme asymmetry levels (0.7), the percent of STSR change decreases significantly, also indicating that a potential saturation level exists.

Previous studies have only compared symmetry changes when inducing symmetry (in patient groups) or asymmetry (in healthy groups) using a single target level [11,12,14,23,30]. However, this study investigated multiple target levels to further understand the symmetry changes at those different targets using the same biofeedback strategy. This is particularly important when RAS gait training is employed for populations with mobility challenges since the findings of this study suggest that setting the metronome to a target beyond the intended target may potentially bring the individual closer to perfect symmetry. For example, lower-limb prosthesis users often exhibit temporal asymmetry, favoring their intact limb over their prosthetic one. Hence, exploring the possibility of setting a higher target (i.e., the metronome is set to an asymmetry toward the prosthetic side) may lead to greater improvements toward symmetry.

When comparing the different strategies, based on the results in Table 6, COL and VOL were comparable to each other. However, VCL yielded significantly different results compared to COL and VOL, where higher changes in the STSR were present with the VCL strategy. This indicates that incremental RAS that is based on an individual’s progress, such as VCL, may be more beneficial to induce changes in the STSR. Furthermore, this aligns with the outcomes presented in the work of Zhang et al., where a wearable biofeedback system was developed to provide real-time rhythmic vibrotactile stimulation to induce changes in walking speed by modulating cadence with 10 healthy individuals [25]. The study revealed that both open- and closed-loop rhythmic stimulations could increase cadence. However, the closed-loop stimulation maintained individuals’ natural gait variability, leading to better adaptations to the stimulation compared to open-loop. Although the study by Zhang et al. controlled cadence using RAS (rather than symmetry, as our study did), the results suggest that the use of closed-loop systems, similar to our findings with VCL, can result in better adaptation. Additionally, the results of the workload index (NASA-TLX) indicated similar workload levels between strategies, which is promising for the use of closed-loop systems, as they do not appear to have resulted in significantly higher workloads.

Furthermore, the results shown in Table 7 provide insights into the delay exhibited by each limb (heel-strike) in response to the metronome cues and whether the lead or lag in response to the metronome cues is symmetrical between the limbs. Significant differences between the right and left legs for COL and VOL were found, whereas the delay was similar comparing the right and left sides for VCL. The differential responses between the right and left legs for COL and VOL highlight the importance of considering limb-specific characteristics (e.g., different gait compensatory strategies on the limb side with induced asymmetry) when implementing RAS-based biofeedback interventions. Understanding such disparities can aid in optimizing the efficacy of the biofeedback system and fine-tuning its application to suit individual limb responses.

One of the challenges associated with other biofeedback strategies (e.g., corrective) is the adverse effect of STSR-based biofeedback on speed and cadence. Escamilla-Nunez et al. compared three different corrective biofeedback strategies (sensory stimulation is provided only when an individual is out of a certain range) to induce TGA, resulting in symmetry changes of up to 0.10 and 0.05 on average for able-bodied individuals [30] and lower-limb amputees [14], respectively. However, the symmetry changes came at a cost, as the average walking speed and cadence significantly decreased by 20% and 15%, respectively [14,30]. In contrast, in this study, cadence and speed remained unchanged while symmetry improved, thus suggesting that RAS strategies may be better suited (over, for example, corrective) for rehabilitation programs aiming to improve overall mobility.

As aforementioned, previous research has primarily focused on utilizing RAS at fixed target levels. Consequently, this study serves as a foundation for exploring potential alternative approaches that adopt a gradual trajectory toward the patient’s desired symmetry goal. The results indicate that there were no potentially negative impacts during gait when continuously varying the symmetry of the metronomes (e.g., VOL and VCL strategies).

Despite the valuable insights gained from this study regarding using RAS-based biofeedback to elicit gait changes, several limitations warrant further investigation and future work. Firstly, it remains unclear whether the observed percent of the STSR changes not reaching 100% is primarily attributed to inaccurate perception of the STSR target or if there exists a saturation level resulting from motor control or biomechanical limitations, for example. Therefore, comprehensive investigations are needed to elucidate the underlying factors contributing to not matching the target levels exactly and to better understand the perceptual and biomechanical aspects of RAS-induced gait changes. Another limitation is that this study only evaluated the immediate short-term effects, while gait (re)training is known to be more effective when performed over longer periods of time. To establish the long-term effectiveness of RAS as a gait rehabilitation tool, it is essential to conduct extended evaluations beyond the intervention phase. Understanding the sustainability of RAS effects and its potential for reducing the frequency of RAS usage after adaptation would provide valuable information for practical application and extended rehabilitation benefits. Moreover, future work should aim to translate the findings of this study to relevant patient populations. Understanding the potential applications and adaptations required for various patient populations will help to refine RAS interventions and address their unique gait-related challenges.

## 5. Conclusions

This work uniquely evaluated a wearable RAS-based biofeedback system with open- and closed-loop strategies. All strategies were effective in altering symmetry during gait while holding cadence and walking speed constant. While symmetries were altered, the target symmetries were not fully reached. This suggests that setting the metronome to a target beyond the intended target may potentially bring the individual closer to the desired symmetry. The outcomes from this study suggest that the use of closed-loop systems (compared to open-loop), can result in better adaptation to changes in temporal symmetry using RAS. None of the strategies significantly differed in terms of user workload, indicating that the continually changing target of closed-loop strategies is not any more taxing than open-loop strategies. This investigation may open up new avenues for optimizing gait training protocols and enhancing the overall effectiveness of interventions for individuals seeking to achieve gait symmetry, namely those based on biofeedback RAS strategies.

## Figures and Tables

**Table 1:** A detailed description of the RAS-based BFB strategies tested.

Strategy	Description
Constant Open-Loop (COL)	The target STSR remained constant throughout the walking trial. Data were collected for the target STSR = 1.0, 0.9, 0.8, and 0.7.
Variable Open-Loop (VOL)	The target STSR decreased by an increment of 0.3–0.4% every full gait cycle.
Variable Closed-Loop (VCL)	The target STSR decreased by an increment of 3–4% only when the participant’s real-time STSR ∈ target STSR ± error.

**Table 2:** Summary of STSR results across all participants (including statistical analysis).

	STSR (Mean ± Std)				BFB vs. Baseline Trials (*p*-Values)			
	Target Cluster				Target Cluster			
Strategy	1	0.9	0.8	0.7	1	0.9	0.8	0.7
COL	0.97 ± 0.02	0.94 ± 0.03	0.90 ± 0.06	0.89 ± 0.09	**0.355**	**0.028**	**0.006**	**0.02**
VOL	0.95 ± 0.04	0.93 ± 0.05	0.90 ± 0.05	0.89 ± 0.05	**0.065**	**0.027**	**<0.001**	**<0.001**
VCL	0.96 ± 0.03	0.91 ± 0.06	0.88 ± 0.06	-	**0.193**	**0.004**	**0.008**	**-**

Statistically significant differences are highlighted in **bold** font (critical alpha value = 0.05).

**Table 3:** Summary of STSR change and percent of STSR change results across all participants for each condition.

	STSR Change			Percent of STSR Change		
Target Cluster	COL	VOL	VCL	COL	VOL	VCL
0.9	0.04 (0.02, 0.07)	0.05 (0.02, 0.09)	0.05 (0.02, 0.13)	59.16 (21.23, 113.58)	53.04 (21.02, 69.99)	68.7 (21.98, 116.94)
0.8	0.08 (0.05, 0.13)	0.07 (0.03, 0.13)	0.10 (0.06, 0.15)	46.71 (24.91, 115.84)	41.67 (18.93, 58.34)	55.17 (32.55, 109.38)
0.7	0.09 (0.05, 0.17)	0.08 (0.05, 0.13)	- *	33.21 (18.23, 125.32)	34.7 (16.96, 48.74)	- *

* Percent change was not calculated for target level 1.0 since it was calculated relative to the difference between the baseline and target (which is close to zero at target level 1.0). Since the percent change data was non-normally distributed, median and interquartile (IQR1, IQR3) values are reported.

**Table 4:** Pairwise comparison between target levels using the STSR and STSR percent errors.

	STSR (*p*-Values)			Percent of STSR Change (*p*-Values)		
Target Pair	COL	VOL	VCL	COL	VOL	VCL
(1.0, 0.9)	**<0.001**	**0.049**	**<0.001**	-	-	-
(1.0, 0.8)	**<0.001**	**<0.001**	**<0.001**	-	-	-
(1.0, 0.7)	**<0.001**	**<0.001**	-	-	-	-
(0.9, 0.8)	**<0.001**	**<0.001**	**<0.001**	0.076	**<0.001**	0.103
(0.9, 0.7)	**<0.001**	**<0.001**	-	**<0.001**	**<0.001**	-
(0.8, 0.7)	0.1831	0.052	-	**<0.001**	**<0.001**	-

Statistically significant differences are highlighted in **bold** font (critical alpha value = 0.05).

**Table 5:** Cadence and speed results across all participants (including statistical analysis).

	Cadence (Steps/min)				Speed (m/s)			
BFB Strategy	1	0.9	0.8	0.7	1	0.9	0.8	0.7
COL	106.6 ± 6.9	106.2 ± 6.5	106.8 ± 6.8	106.3 ± 6.4	1.2 ± 0.15	1.19 ± 0.14	1.19 ± 0.13	1.16 ± 0.13
VOL	106.4 ± 6.9	106.4 ± 6.9	106.4 ± 6.9	105.4 ± 6.5	1.17 ± 0.14	1.15 ± 0.14	1.13 ± 0.14	1.14 ± 0.17
VCL	106.4 ± 6.9	106.5 ± 6.9	105.4 ± 6.5	- **	1.18 ± 0.15	1.15 ± 0.14	1.12 ± 0.13	- **
	**Pairwise Comparison (*p*-values)**				**Pairwise Comparison (*p*-values)**			
COL	- *	0.885	0.953	0.919	- *	0.941	0.916	0.580
VOL	0.945	0.946	0.946	0.948	0.726	0.501	0.386	0.574
VCL	0.952	0.957	0.956	- **	0.846	0.557	0.403	- **

* A pairwise comparison was conducted relative to the cadence and speed for COL at target level 1. ** Cadence was not calculated for target level 1.0 since it was calculated relative to the difference between baseline and target (which is close to zero at target level 1.0).

**Table 6:** Pairwise comparison between strategies using the STSR and percent of STSR change.

	STSR (*p*-Values)			Percent of STSR Change (*p*-Values)		
Target Cluster	COL–VOL	COL–VCL	VOL–VCL	COL–VOL	COL–VCL	VOL–VCL
1	**0.004**	**0.028**	0.285	-	-	-
0.9	0.469	**0.002**	**0.005**	0.331	**0.004**	**0.049**
0.8	0.481	**<0.001**	**<0.001**	0.638	**<0.001**	**<0.001**
0.7	0.34	-	-	0.474	-	-

Statistically significant differences are highlighted in bold font (critical alpha value = 0.05).

**Table 7:** Time error (difference between heel-strike and beat sound) results.

Limb Side	Time Error (ms) (Mean ± Std)			Pairwise Comparison (Right vs. Left Side)		
	COL	VOL	VCL	COL	VOL	VCL
Right	−88.9 ± 57.4	−137.3 ± 36.8	−146.1 ± 19.4	**0.0309**	**0.0436**	0.2219
Left	−119.8 ± 70.7	−174.1 ± 47.1	−173.4 ± 50.1			

Statistically significant differences are highlighted in bold font (critical alpha value = 0.05).

**Table 8:** NASA-TLX results (scores and statistical analysis comparison between each strategy).

	NASA-TLX Score (Mean ± Std)			Pairwise Comparison (*p*-Values)		
Factor	COL	VOL	VCL	COL–VOL	VOL–VCL	COL–VCL
Mental Demand	44.5 ± 24.11	45.5 ± 26.84	55.5 ± 24.68	0.8760	0.0548	0.1599
Physical Demand	36 ± 18.44	35.5 ± 23	40 ± 22.48	0.9151	0.2042	0.4383
Temporal Demand	31.5 ± 19.47	36.5 ± 19.81	38.5 ± 20.89	0.4997	0.7509	0.1162
Performance	40.5 ± 30.84	34.5 ± 20.18	47.5 ± 24.84	0.3399	0.1186	0.2977
Effort	45 ± 25.73	51 ± 29.11	50 ± 25.8	0.3092	0.8363	0.4433
Frustration	25 ± 20.05	26 ± 20.23	27.5 ± 15.7	0.7435	0.7993	0.7080
Overall Score	42.33 ± 16.22	46.97 ± 16.26	49.43 ± 14.31	0.6194	0.3280	0.1558

## Data Availability

The data presented in this study are available on request from the corresponding author. The data are not publicly available due to ethics restrictions on data dissemination and storage.
